# Enpp1 is an anti-aging factor that regulates Klotho under phosphate overload conditions

**DOI:** 10.1038/s41598-017-07341-2

**Published:** 2017-08-10

**Authors:** Ryuichi Watanabe, Nobuyuki Fujita, Yuiko Sato, Tami Kobayashi, Mayu Morita, Takatsugu Oike, Kana Miyamoto, Makoto Kuro-o, Toshimi Michigami, Seiji Fukumoto, Takashi Tsuji, Yoshiaki Toyama, Masaya Nakamura, Morio Matsumoto, Takeshi Miyamoto

**Affiliations:** 1 Department of Orthopedic Surgery, Keio University School of Medicine, 35 Shinano-machi, Shinjuku-ku, Tokyo, 160-8582 Japan; 2 Department of Advanced Therapy for Musculoskeletal Disorders, Keio University School of Medicine, 35 Shinano-machi, Shinjuku-ku, Tokyo, 160-8582 Japan; 3 Department of Musculoskeletal Reconstruction and Regeneration Surgery, Keio University School of Medicine, 35 Shinano-machi, Shinjuku-ku, Tokyo, 160-8582 Japan; 4 Division of Oral and Maxillofacial Surgery, Department of Dentistry and Oral Surgery, Keio University School of Medicine, 35 Shinano-machi, Shinjuku-ku, Tokyo, 160-8582 Japan; 5 Center for Molecular Medicine, Jichi Medical University, Shimotsuke, Tochigi, 329-0498 Japan; 6 Department of Bone and Mineral Research, Osaka Medical Center and Research Institute for Maternal and Child Health, Izumi, Osaka, 594-1101 Japan; 7 Fujii Memorial Institute of Medical Sciences, Tokushima University, Tokushima, Tokushima, 770-8503 Japan

## Abstract

Control of phosphate metabolism is crucial to regulate aging in mammals. Klotho is a well-known anti-aging factor that regulates phosphate metabolism: mice mutant or deficient in *Klotho* exhibit phenotypes resembling human aging. Here we show that ectonucleotide pyrophosphatase/phosphodiesterase 1 (Enpp1) is required for Klotho expression under phosphate overload conditions. Loss-of-function *Enpp1* ^*ttw/ttw*^ mice under phosphate overload conditions exhibited phenotypes resembling human aging and Klotho mutants, such as short life span, arteriosclerosis and osteoporosis, with elevated serum 1,25(OH)_2_D_3_ levels. *Enpp1* ^*ttw/ttw*^ mice also exhibited significantly reduced renal *Klotho* expression under phosphate overload conditions, and aging phenotypes in these mice were rescued by Klotho overexpression, a low vitamin D diet or vitamin D receptor knockout. These findings indicate that Enpp1 plays a crucial role in regulating aging via Klotho expression under phosphate overload conditions.

## Introduction

A fundamental question in human biology is what mechanisms underlie aging. Aging is reportedly defined as age-related deterioration of physiological functions necessary for survival and fertility^1^. Several phenotypes associated with aging have been identified, such as short life span, osteoporosis, arteriosclerosis, cancer and cataract development. To date, various mouse models showing premature aging syndromes have been established and characterized, such as Klotho (*kl/kl*)-^2^, SAM-^3^, ATM-^4^, p53-^5^, FoxOs-^6^, telomerase^7^-, and Fetuin A-deficient^8^ mice; however, none exhibits the full range of aging phenotypes.

Among models of aging, the *kl/kl* mouse is best known for showing premature aging phenotypes including short life span, osteoporosis and arteriosclerosis^2^. Klotho is a transmembrane protein that gives rise to a soluble form when cleaved at the cell surface. The transmembrane Klotho (αKlotho) forms a heterocomplex with fibroblast growth factor receptors (FGFRs) required for high affinity FGF23 binding and signaling^9–11^. FGF23 gain-of-function mutations reportedly underlie autosomal dominant hypophosphatemic rickets (ADHR)^12, 13^, evidence that FGF23 regulates phosphate metabolism. FGF23-deficient mice reportedly exhibit aging-related phenotypes similar to *kl/kl* mice^14^, suggesting that FGF23 and Klotho co-operate in regulating phosphate metabolism. Klotho and FGF23 are reported required to regulate circulating 1,25(OH)_2_D_3_ levels by suppressing expression of *cyp27b1* in the kidney^15, 16^. Indeed, premature aging phenotypes seen in either *kl/kl* or FGF23-deficient mice are completely rescued by ablation of the Vitamin D receptor (VDR)^17–19^. Loss-of-function mutations in either *KLOTHO* or *FGF23* reportedly cause tumoral calcinosis in humans, a disease characterized by ectopic, vascular calcifications^20, 21^. FGF23 is mainly produced in bone^5^, and FGF23 secretion in bone is stimulated by 1,25(OH)_2_D_3_ and by increased extracellular phosphate, an activity regulated in a feedback loop between bone and kidney^22, 23^. In contrast, *Klotho* expression is downregulated by phosphate^24^ and reportedly regulated epigenetically^25^. However, mechanisms underlying *Klotho* regulation are unclear, and there are as yet no animal models resembling *kl/kl* mice that have been established by deleting factors regulating *Klotho*.

Ectonucleotide pyrophosphatase/phosphodiesterase 1 (Enpp1) is a single-pass transmembrane protein and major generator of extracellular pyrophosphate (PPi), which inhibits hydroxyapatite crystal deposition^26^. *ENPP1* mutations cause autosomal recessive hypophosphatemic rickets (ARHR) or Generalized Arterial Calcification of Infancy (GACI) in humans^27–30^. ARHR caused either by *ENPP1* mutation or mutations in *PHEX* or *DMP1* genes is marked by high circulating FGF23 levels^31, 32^. In contrast, GACI is a rare autosomal-recessive disorder characterized by calcification of diffuse vascular and periarticular soft-tissues, and few GACI patients survive the neonatal period^33^. *ENPP1* mutations are also seen in patients with ossification of the posterior longitudinal ligament (OPLL), a disease characterized by ectopic ossification in a spinal ligament, although mechanisms are unknown^34–36^. The *Enpp1* mutants *Enpp1* ^*ttw/ttw*^ or *Enpp1* ^*asj/asj*^ reportedly exhibit OPLL- or GACI-like, respectively, phenotypes^37, 38^.

Here, we show that Enpp1 acts as an anti-aging factor under phosphate overload by regulating Klotho expression. *Enpp1* ^*ttw/ttw*^ or genetically engineered *Enpp1*-deleted mice (*Enpp1* ^*Δ/Δ*^) exhibited premature aging phenotypes, such as short life span and arteriosclerosis, phenotypes resembling *kl/kl* mice and human aging, under phosphate overload. *Klotho* expression in kidney was significantly downregulated in *Enpp1* ^*ttw/ttw*^ mice by phosphate overload, and premature aging phenotypes seen in *Enpp1* ^*ttw/ttw*^ mice under overload conditions were completely rescued by VDR ablation. Thus, Enpp1-Klotho-VDR signals are required to prevent premature aging phenotypes, particularly under phosphate overload conditions.

## Results

### *Enpp1* mutation causes premature aging phenotypes under phosphate overload

**Figure 1:**
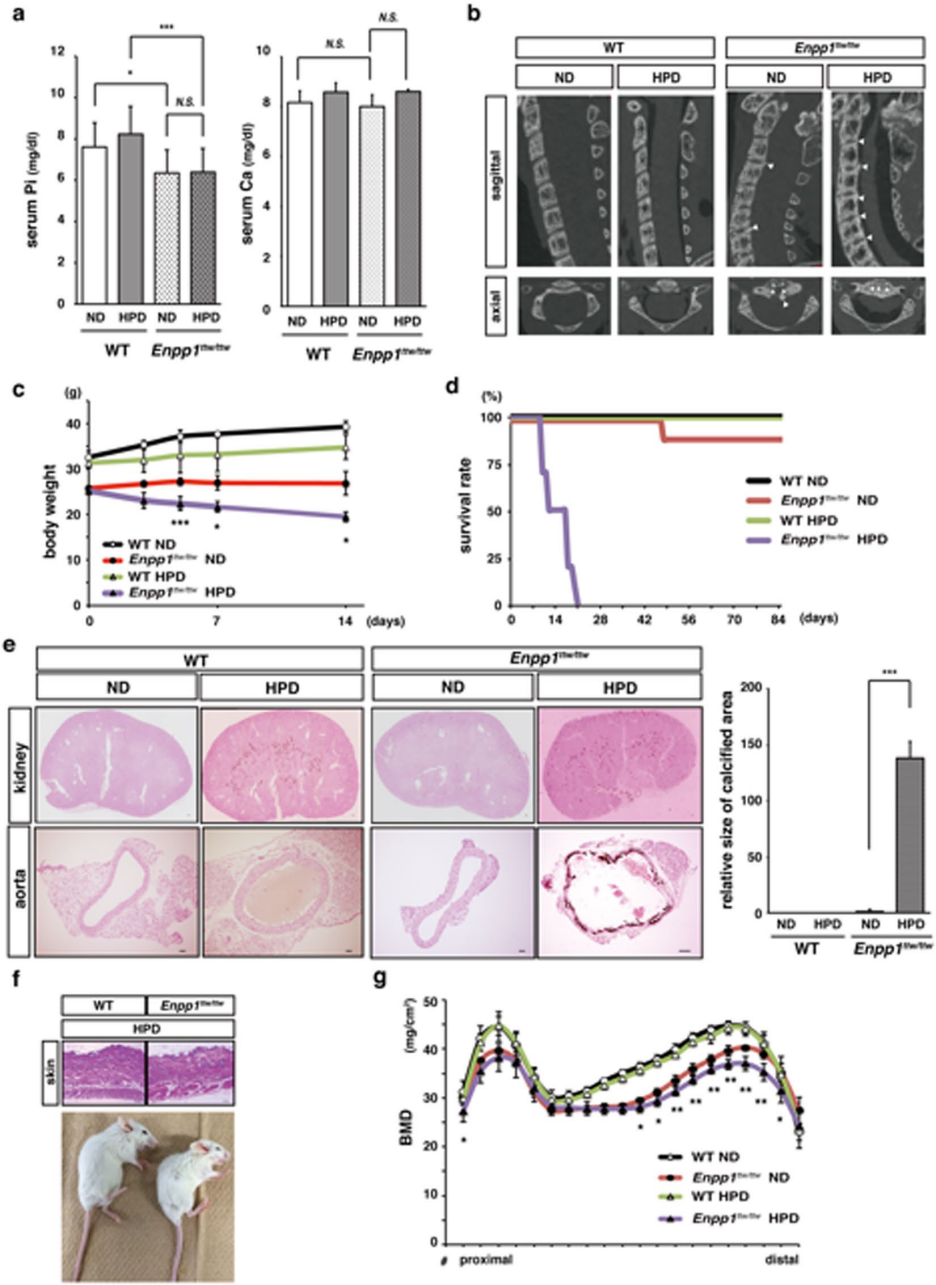
A high phosphate diet promotes premature aging phenotypes in *Enpp1* ^*ttw/ttw*^ mice. Eight-week-old wild-type and *Enpp1* ^*ttw/ttw*^ mice were fed a normal (ND) or high phosphate (HPD) diet for two (**a**–**c**, **e–** **g**) to twenty weeks (**d**). The following parameters were then analyzed: serum phosphate and calcium levels (**a**); ectopic calcification at the posterior longitudinal ligaments and intervertebral discs micro-computed tomography (**b**); body weight changes (**c**); survival rate (each group; n = 10) (**d**); ectopic calcification in kidney and aorta by von Kossa staining (**e**); skin atrophy by HE staining (upper, f); gross appearance (lower, f); and bone mineral density (BMD) of femurs equally divided longitudinally by DEXA (**g**) after feeding a HPD. Data represent mean indicated parameters ± S.D. (^*^ *p* < 0.05; ^**^ *p* < 0.01; ^***^ *p* < 0.001; NS, not significant; *n* = 5 or 6; c and g, *Enpp1* ^*ttw/ttw*^ mice fed a ND vs HPD). Arrowheads in (**b**) represent OPLL formation. Bar = 100 μm (**e**). Representative data of at least two independent experiments are shown.

*Enpp1* mutations cause several human diseases, among them AHRH, GACI or OPLL^12–15^. *Enpp1* ^*ttw/ttw*^ mice are *Enpp1* mutation mouse models that exhibit OPLL-like phenotypes^38^. We confirmed that at 8 weeks of age *Enpp1* ^*ttw/ttw*^ mice show significantly lower serum phosphate (Pi) levels relative to controls, an outcome also seen in ARHR patients (Fig. 1a). Although not statistically significant, serum calcium levels were elevated in both WT or *Enpp1* ^*ttw/ttw*^ mice by a high phosphate diet (HPD) (Fig. 1a). However, feeding 8-week old *Enpp1* ^*ttw/ttw*^ mice a HPD did not elevate serum phosphate levels in as it did in wild-type (WT) mice (Fig. 1a). Ectopic calcification in the ear was previously demonstrated to be accelerated by feeding *Enpp1* ^*ttw/ttw*^ mice a HPD^39^. We found that HPD worsened OPLL phenotypes, as shown by elevated ectopic calcification in the posterior longitudinal ligament (PLL) of *Enpp1* ^*ttw/ttw*^ mice (Fig. 1b). Moreover, after phosphate overload, *Enpp1* ^*ttw/ttw*^ mice showed body weight loss (Fig. 1c), became inactive and marantic, and died within three weeks (Fig. 1d). *Enpp1* ^*ttw/ttw*^ mice fed a phosphate diet also exhibited ectopic calcification in aorta and kidney (Fig. 1e), atrophic skin (Fig. 1f) and osteoporotic reduced bone mass (Fig. 1g), all premature aging phenotypes seen in *kl/kl* mice and aging humans^2^.

*Enpp1* ^*ttw/ttw*^ mice showed calcification in kidney and aorta at cellular levels (Supplementary Fig. 1a). Expression of runt-related transcription factor 2 (Runx2), a factor essential for osteoblastogenesis^40^, was significantly upregulated in aorta of *Enpp1* ^*ttw/ttw*^ mice fed a HPD rather than a normal diet (Supplementary Fig. 1b), suggesting that ectopic ossification is likely due to trans-differentiation of cells into an osteoblastic lineage. Although, no differences were detected in senescence-associated beta-galactosidase (SA *β-*gal) staining, expression of p16, another aging-related factor, increased in kidney of *Enpp1* ^*ttw/ttw*^ mice fed a HPD relative to that of *Enpp1* ^*ttw/ttw*^ mice fed a ND or wild-type (WT) mice fed either diet (Supplementary Fig. 2).

We also found that serum levels of receptor activator of nuclear factor kappa B ligand (RANKL), a cytokine essential for osteoclastogenesis^41^, increased in *Enpp1* ^*ttw/ttw*^ mice fed a HPD compared with *Enpp1* ^*ttw/ttw*^ mice fed a ND or WT mice fed either diet (Supplementary Fig. 3a), but serum osteoprotegerin (OPG), a natural agonist of RANKL^42^, also increased (Supplementary Fig. 3b), and as a result, the serum RANKL/OPG ratio was comparable in *Enpp1* ^*ttw/ttw*^ mice fed a HPD relative to *Enpp1* ^*ttw/ttw*^ mice fed a ND or WT mice fed either diet (Supplementary Fig. 3c). However, osteoclast bone resorbing activity, as measured by serum CTx levels, was significantly elevated in *Enpp1* ^*ttw/ttw*^ mice fed a HPD, suggesting that decreased bone mineral density is due at least in part to elevated osteoclast bone-resorption (Supplementary Fig. 3d).

Although statistically not significant, serum creatinine and blood urinary nitrogen (BUN) levels were elevated in *Enpp1* ^*ttw/ttw*^ mice fed a HPD relative to *Enpp1* ^*ttw/ttw*^ mice fed a ND or WT mice fed either diet, and urine volume was significantly decreased in *Enpp1* ^*ttw/ttw*^ mice fed a HPD compared to WT mice fed a HPD diet (Supplementary Fig. 4a and b), suggesting that *Enpp1* ^*ttw/ttw*^ mice fed a HPD undergo renal dysfunction. Urinary calcium was elevated by HPD in WT mice, a phenotype not seen in *Enpp1* ^*ttw/ttw*^ mice fed a HPD (Supplementary Fig. 4c). In contrast, urinary phosphate and creatinine levels were elevated and downregulated, respectively, in both *Enpp1* ^*ttw/ttw*^ and WT mice by HPD (Supplementary Fig. 4c).

**Figure 2:**
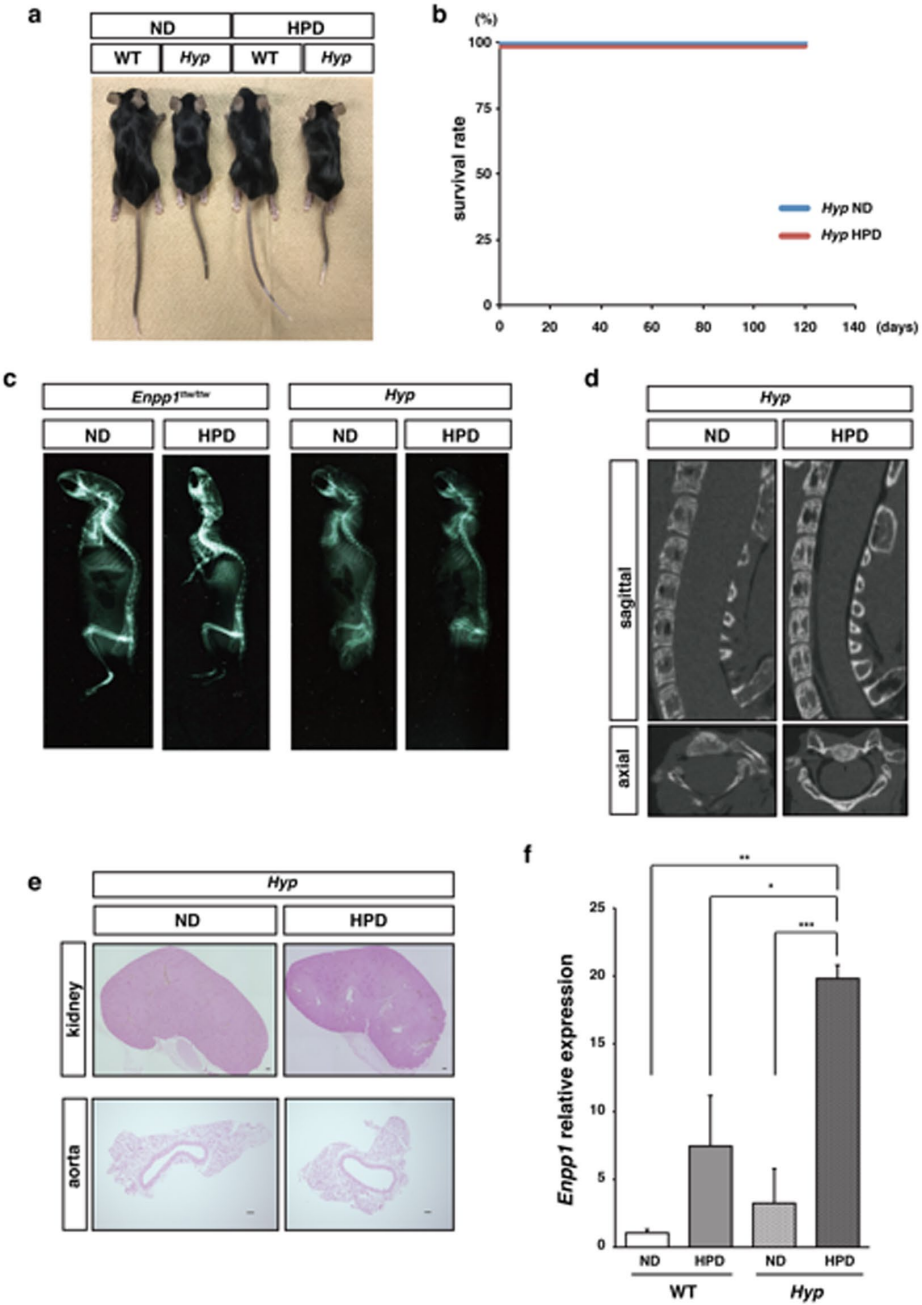
Hyp mice fed a high phosphate diet do not exhibit aging phenotypes. Eight-week-old wild-type or Hyp mice were fed with normal (ND) or high phosphate (HPD) diet for eight weeks. Mice were then analyzed for: gross appearance (**a**); survival rate (each group; n = 6) (**b**); soft X-ray images of total spine (**c**); ectopic calcification around vertebral bones by micro-computed tomography (**d**); ectopic calcification in kidney and aorta by von Kossa staining. Bar = 100 μm (**e**); and *Enpp1* expression in femoral bones by realtime PCR (**f**). Data in (**f**) represents mean *Enpp1* expression relatibe to *β-actin* ± S.D. (^*^ *p* < 0.05; ***p < 0.001; *ns*, not significant; *n* = 6). Representative data of at least two independent experiments are shown.

In contrast, Hyp mice, a different model of hypophosphatemic rickets caused by mutation in the *Phex* gene, did not exhibit premature aging or OPLL phenotypes even when fed a HPD (Fig. 2). Eight-week-old Hyp mice were fed a HPD for two weeks, but since mice exhibited no obvious phenotypes, feeding of the HPD was extended six weeks longer (Fig. 2). HPD effectively elevated serum phosphate levels in these Hyp mice (data not shown), as previously described^43^, an effect not seen in *Enpp1* ^*ttw/ttw*^ mice fed the same diet for two weeks (Fig. 1a). Unlike *Enpp1* ^*ttw/ttw*^ mice, which exhibited visible aging phenotypes after two weeks of phosphate overload, Hyp mice were normal in appearance (Fig. 2a), nor did they exhibit lethality after eight weeks of phosphate overload (Fig. 2b). Soft X-ray images showed that *Enpp1* ^*ttw/ttw*^ showed kyphosis by two weeks of HPD feeding, but Hyp mice did not become kyphotic even after eight weeks of phosphate overload (Fig. 2c). Hyp mice at eight weeks of phosphate overload did not show OPLL, renal or aortic calcification (Fig. 2d and e). *Enpp1* expression in kidney was significantly higher in Hyp than in WT mice and was significantly upregulated by HPD in mice of either genotype (Fig. 2f). These results suggest that in mice, premature aging phenotypes associated with phosphate overload are not common to all cases of hypophosphatemic rickets mice but rather they require mutation in *Enpp1*.

### *Enpp1*^*ttw/ttw*^ mice under phosphate overload show reduced Klotho expression

**Figure 3:**
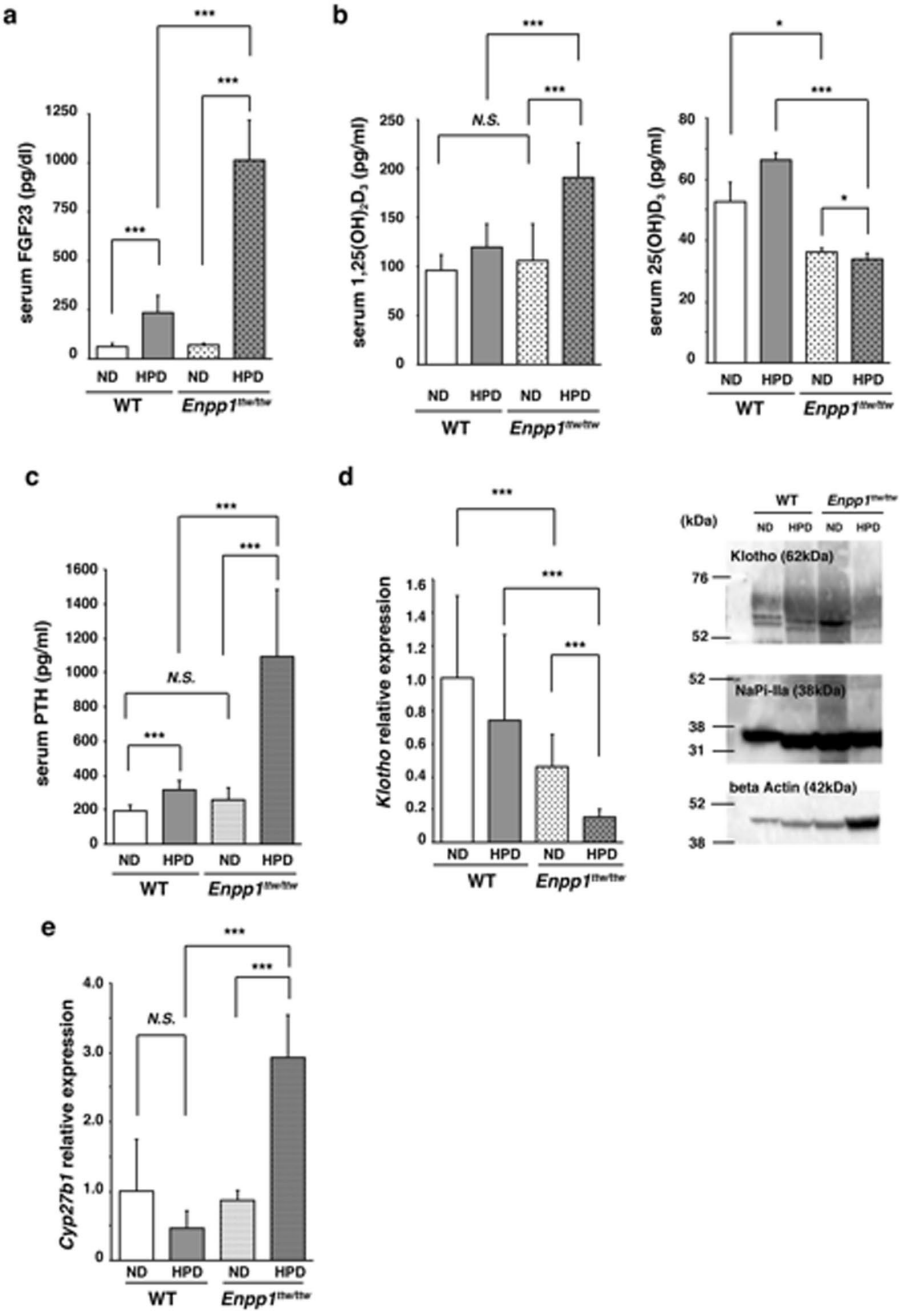
Dietary phosphate overload decreases Klotho expression in kidney of *Enpp1* ^*ttw/ttw*^ mice. Eight-week-old wild-type and *Enpp1* ^*ttw/ttw*^ mice were fed a ND or HPD for two weeks, and serum levels of FGF23 (**a**), 1,25(OH)_2_D_3_ (b, left panel), 25(OH)D_3_ (b, right panel) or PTH (**c**) were analyzed. Klotho expression in kidney was analyzed by realtime PCR (d, left panel) and western blot (d, right panel). NaPi-IIa expression was also analyzed by western blot (d, right panel). *Cyp27b1* expression in kidney was also analyzed by realtime PCR (**e**). Data in (**a**), (**b**) and (**c**) represent mean values of the indicated parameter ± S.D. (*p < 0.05; ***p < 0.001; *ns*, not significant; *n* = 6). Data in (**d**) and (**e**) represent mean *Klotho* or *Cyp27b1* expression relative to *β-actin* ± SD (*p < 0.05; ***p < 0.001; *ns*, not significant; *n* = 6). Actin serves as an internal control (**c**). Representative data of at least three independent experiments are shown.

Aging phenotypes seen in *Enpp1* ^*ttw/ttw*^ mice fed a HPD, such as short lifespan and ectopic calcifications, resemble those seen in *kl/kl* mice, and *kl/kl* mice reportedly exhibit abnormal correlation between serum FGF23 and 1,25(OH)_2_D_3_ levels^44^. Moreover, both factors are known to regulate serum phosphate levels. Thus, we fed eight-week-old wild-type or mutant mice a normal or HPD for two weeks, since *Enpp1* ^*ttw/ttw*^ mice fed a HPD died within three weeks of feeding (Fig. 1), and then analyzed both for serum FGF23 and active vitamin D3, 1,25(OH)_2_D_3_, levels. Both were upregulated significantly in *Enpp1* ^*ttw/ttw*^ compared with control mice under phosphate overload (Fig. 3a and b). 1,25(OH)_2_D_3_ is derived from 25(OH)D_3_ due to hydroxylation by the enzyme cyp27b1^45^. In accordance, serum 25(OH)D_3_ levels were significantly lower in *Enpp1* ^*ttw/ttw*^ mice fed a HPD compared with those fed a normal diet or wild-type mice fed either diet (Fig. 3b). Serum PTH levels were also significantly elevated in WT or *Enpp1* ^*ttw/ttw*^ mice fed a HDP (Fig. 3c). FGF23 is known to down-regulate 1,25(OH)_2_D_3_^14^; thus it is unusual that FGF23 and 1,25(OH)_2_D_3_ levels would be concomitantly elevated, as seen in *Enpp1* ^*ttw/ttw*^ mice. However, *Klotho* mutant mice reportedly show elevated levels of both FGF23 and 1,25(OH)_2_D_3_ in sera^2^, suggesting that *Klotho* expression is suppressed in *Enpp1* ^*ttw/ttw*^ mice under phosphate overload. Furthermore, since aging phenotypes in *Enpp1* ^*ttw/ttw*^ mice under phosphate overload phenocopy *kl/kl* mice, we next analyzed *Klotho* expression in *Enpp1* ^*ttw/ttw*^ mouse kidney (Fig. 3d and Supplementary Fig. 5). As expected, renal *Klotho* mRNA and protein expression were significantly lower in *Enpp1* ^*ttw/ttw*^ than in wild-type mice under phosphate overload conditions (Fig. 3d and Supplementary Fig. 5). Klotho reportedly antagonizes expression of the sodium-phosphate co-transporter NaPi-IIa, also called solute carrier family 34 (sodium phosphate), member 1 (Slc34a1)^46^. Indeed, we observed elevated NaPi-IIa expression inversely correlated with Klotho expression in *Enpp1* ^*ttw/ttw*^ mice fed a HPD (Fig. 3d and Supplementary Fig. 5). Furthermore, Klotho is reportedly required to inhibit *cyp27b1* ^47^. We found that *cyp27b1* expression in kidney was significantly upregulated in *Enpp1* ^*ttw/ttw*^ mice fed a HPD compared with those fed a normal diet (Fig. 3d), potentially due to inhibited Klotho expression.

To confirm that aging phenotypes seen in *Enpp1* ^*ttw/ttw*^ mice following phosphate overload are due to reduced *Klotho* expression, we crossed the *Enpp1* ^*ttw/ttw*^ mice with *Klotho*-overexpressing transgenic (*Klotho* Tg) mice, an approach that reportedly rescues aging phenotypes in *kl/kl* mice^2^ (Supplementary Fig. 6). In resultant mice fed a HPD, shortened life span was rescued in part (Supplementary Fig. 6a), while ectopic calcification in aorta was significantly rescued compared to similarly fed *Enpp1* ^*ttw/ttw*^ mice (Supplementary Fig. 6b). These results support the idea that under phosphate overload, decreased Klotho expression due to *Enpp1* mutation promotes development of aging phenotypes in *Enpp1* ^*ttw/ttw*^ mice. We also observed OPLL-like peri-vertebral bone phenotypes in *kl/kl* mice (Supplementary Fig. 7), as is seen in *Enpp1* ^*ttw/ttw*^ mice (Fig. 1b), suggesting that the Enpp1-Klotho axis is required to prevent ectopic calcification and aging phenotypes under phosphate overload conditions.

### Elevated vitamin D levels promote aging phenotypes in *Enpp1*^*ttw/ttw*^ mice under phosphate overload

**Figure 4:**
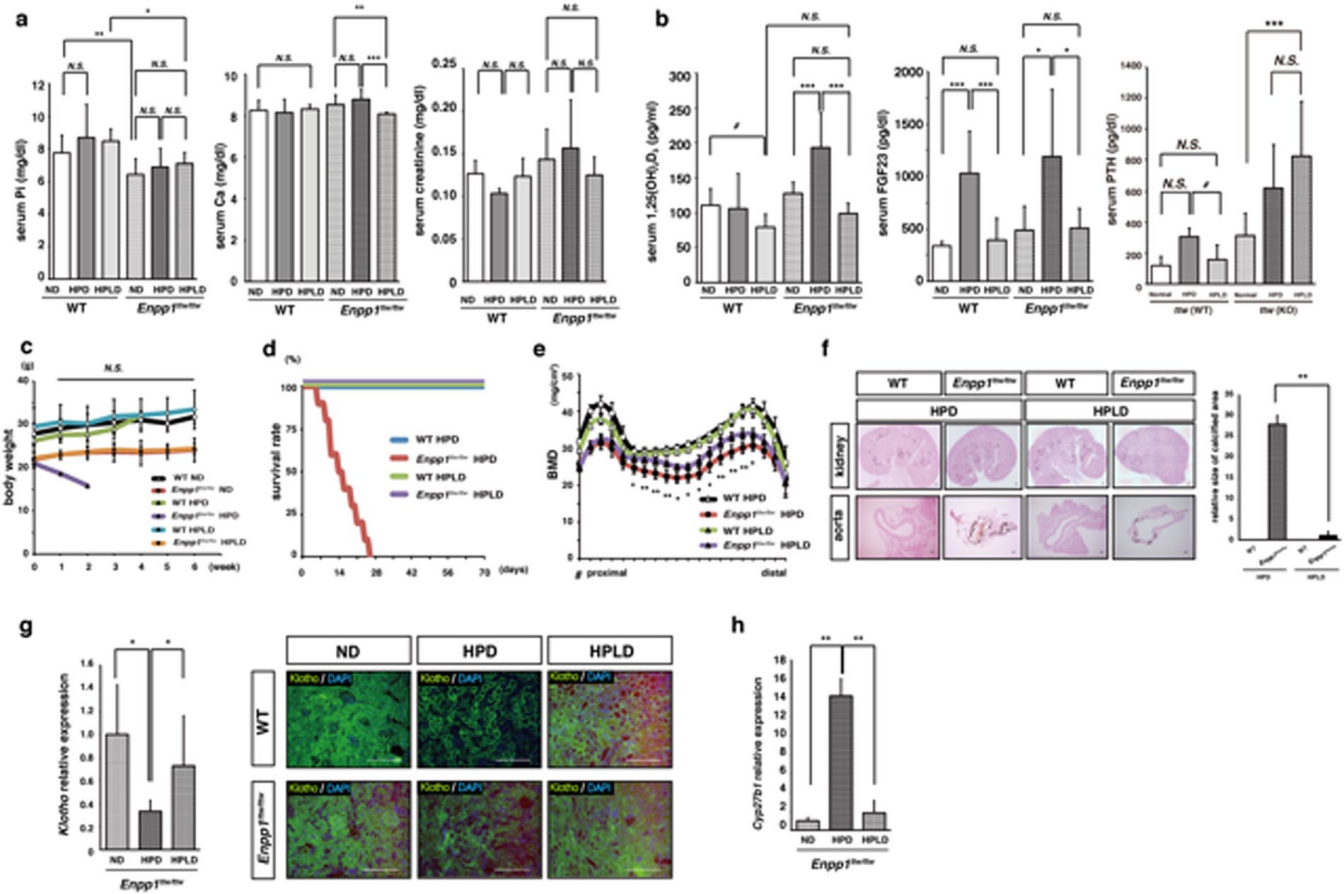
A low vitamin D diet antagonizes aging phenotypes seen in *Enpp1* ^*ttw/ttw*^ mice under phosphate overload. Eight-week-old wild-type and *Enpp1* ^*ttw/ttw*^ mice were fed a ND, HPD or a high phosphate/low vitamin D diet (HPLD) for eight weeks (**a**, **b**, **e**–**h**) or the indicated periods **(c** and **d**). The following parameters were then analyzed: serum levels of phosphorus, calcium and creatinine (**a**); 1,25(OH)_2_D_3_, FGF23 and PTH (**b**); body weight changes (each group; n = 10) (**c**); and survival rate (each group; n = 6) (**d**); bone mineral density (BMD) of femurs equally divided longitudinally by DEXA (**e**); ectopic calcification in kidney and aorta by von Kossa staining (f, left panel); and scoring of calcification area in aorta (f, right panel). *Klotho* expression in kidney was analysed by realtime PCR (g, left panel) and immnunohistological staining (g, right panel). (**h**) mRNA *Cyp27b1* expression level in kidney was analyzed by realtime PCR. Data (**a**, **b**, **e** and **f**) represent mean values of the indicated parameter ± S.D. (#, *p* < 0.1; *p < 0.05; **p < 0.01; ***p < 0.001; *ns*, not significant; each *n* = 6, g *Enpp1* ^*ttw/ttw*^ mice fed a HPD vs HPLD). Data in (**g**) represent mean *Klotho* or *Cyp27b1* expression relative to *β-actin* ± SD (*p < 0.05; *n* = 6). Representative data of at least two independent experiments are shown.

Serum 1,25(OH)_2_D_3_ levels were significantly elevated in *Enpp1* ^*ttw/ttw*^ mice fed a HPD (Fig. 1a), and high 1,25(OH)_2_D_3_ levels reportedly cause premature aging phenotypes in *kl/kl* mice^14^. Thus, we asked whether elevated serum 1,25(OH)_2_D_3_ levels promote aging phenotypes in *Enpp1* ^*ttw/ttw*^ mice under phosphate overload conditions (Fig. 4). To do so, we fed *Enpp1* ^*ttw/ttw*^ mice a high phosphate/reduced vitamin D diet (HPLD) starting at 8 weeks of age (Fig. 4). Compared with animals fed a HPD only, serum phosphate and calcium levels were down-regulated in *Enpp1* ^*ttw/ttw*^ mice fed a high phosphate/low vitamin D (HPLD) diet (Fig. 4a). Urinary calcium and creatinine levels were elevated in both WT and *Enpp1* ^*ttw/ttw*^ mice fed a HPLD compared with those fed a HPD (Supplementary Fig. 8). Urinary phosphate levels were comparable in WT and *Enpp1* ^*ttw/ttw*^ mice fed a HPLD (Supplementary Fig. 8). Serum 1,25(OH)_2_D_3_ and FGF23 levels were down-regulated in both WT or *Enpp1* ^*ttw/ttw*^ mice fed a HPLD (Fig. 4b). Moreover, serum PTH levels were down- and up-regulated in WT and *Enpp1* ^*ttw/ttw*^ mice, respectively, fed a HPLD (Fig. 4b). Reduced body weight, shortened life span, reduced bone mass and ectopic calcification in kidney and aorta, all seen in *Enpp1* ^*ttw/ttw*^ mice fed a HPD, were all significantly rescued by vitamin D depletion, even under high phosphate loading (Fig. 4c–f). Inactivity and maranic phenotypes were prevented in *Enpp1* ^*ttw/ttw*^ mice by depletion of vitamin D from a HPD, suggesting that premature aging phenotypes observed in *Enpp1* ^*ttw/ttw*^ mice under phosphate overload require high vitamin D signals, as is the case with *kl/kl* mice. Reduced *Klotho* expression in kidney seen following phosphate overload in *Enpp1* ^*ttw/ttw*^ mice was significantly rescued by vitamin D depletion (Fig. 4g), suggesting feedback between Klotho and vitamin D signals. Furthermore, correlated with elevated renal *Klotho* expression, *cyp27b1* expression was significantly downregulated by vitamin D depletion under phosphate overload conditions in *Enpp1* ^*ttw/ttw*^ mice (Fig. 4g).

Fetuin A activity antagonizes ectopic calcification, and circulating Fetuin A levels are down-regulated with age^48^. Thus, we performed ELISA analysis to measure Fetuin A levels in WT or *Enpp1* ^*ttw/ttw*^ mice fed a HPD (Supplementary Fig. 9). Serum Fetuin A protein levels significantly decreased in response to a HPD in both genotypes and were equivalent in each (Supplementary Fig. 9). Although aging phenotypes such as short lifespan were rescued in response to a HPLD, low Fetuin A levels were not (Supplementary Fig. 9). Thus, aging phenotypes seen in *Enpp1* ^*ttw/ttw*^ mice fed a HPD are more likely associated with vitamin D rather than with Fetuin A signaling.

**Figure 5:**
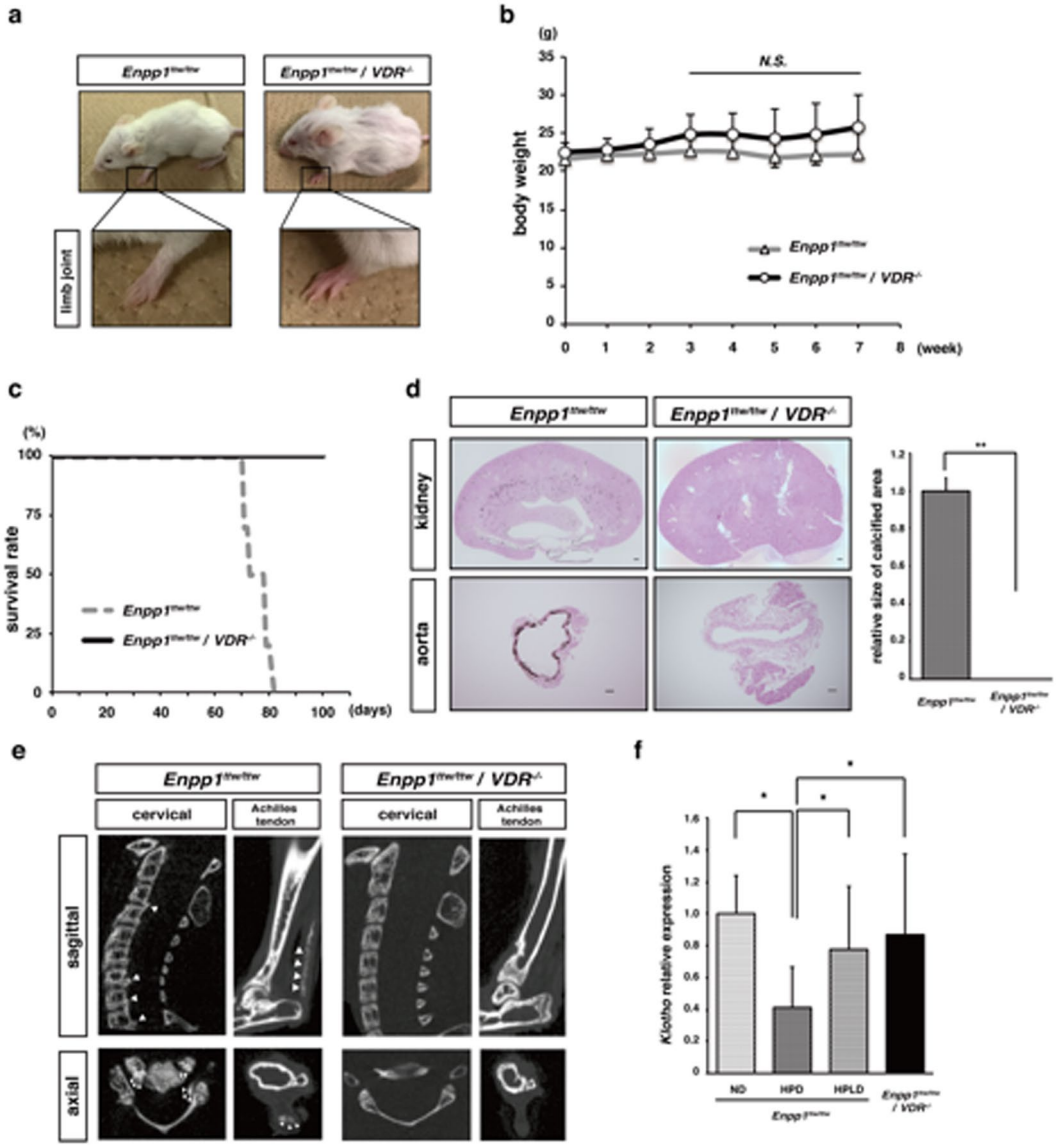
Aging phenotypes seen in *Enpp1* ^*ttw/ttw*^ mice fed a high phosphate diet are abrogated by deletion of vitamin D receptor. Eight-week-old *Enpp1* ^*ttw/ttw*^ or *Enpp1* ^*ttw/ttw*^/*VDR* ^*−/−*^ mice were fed a HPD for indicated periods (**a** and **b**) or eight weeks (**c**–**e**). The following parameters were then analyzed: gross appearance and forelimb shape (**a**); body weight changes (**b**); survival rate (each group; n = 5) (**c**); ectopic calcification in kidney and aorta by von Kossa staining (d, left panel); scoring of the calcification area in aorta (d, right panel); ectopic calcification around vertebral bones by micro-computed tomography (**e**); and *Klotho* expression in kidney by realtime PCR (**f**). Data (**d**) represent mean ectopic calcification area ± S.D. (**p < 0.01; *n* = 6). Data (**e**) represent mean *Klotho* expression relative to *β-actin* ± SD (^*^ *p* < 0.05; *n* = 6). Representative data of at least two independent experiments are shown.

Next we crossed *Enpp1* ^*ttw/ttw*^ with VDR-deficient (*VDR* ^*−/−*^) mice to yield Enpp1/VDR doubly-deficient (*Enpp1* ^*ttw/ttw*^/*VDR* ^*−/−*^) mice (Fig. 5). *VDR* ^*−/−*^ mice die after weaning, but can survive beyond weaning if fed a high calcium diet^49, 50^. Thus, we fed *Enpp1* ^*ttw/ttw*^/*VDR* ^*−/−*^ mice a high phosphate/high calcium diet starting at 8-weeks of age. Others have reported that calcium and phosphate overloading worsens OPLL phenotypes in *Enpp1* ^*ttw/ttw*^ mice^39^. However, ankylosis of the fore- and hindlimb seen in *Enpp1* ^*ttw/ttw*^ mice was efficiently rescued in *Enpp1* ^*ttw/ttw*^/*VDR* ^*−/−*^ mice fed a high phosphate/high calcium diet (Fig. 5a). Reduced body weight, shortened life span, ectopic calcification in kidney and aorta and ossification in ligaments seen in *Enpp1* ^*ttw/ttw*^ mice were all completely abrogated in *Enpp1* ^*ttw/ttw*^/*VDR* ^*−/−*^ mice even under phosphate overload and high calcium conditions (Fig. 5b–e). Moreover, *Enpp1* ^*ttw/ttw*^/*VDR* ^*−/−*^ mice were active and not maranic compared with *Enpp1* ^*ttw/ttw*^ mice. In addition, *Klotho* expression in kidney was significantly high in *Enpp1* ^*ttw/ttw*^/*VDR* ^*−/−*^ relative to *Enpp1* ^*ttw/ttw*^ mice under phosphate overload (Fig. 5f).

## Discussion

**Figure 6:**
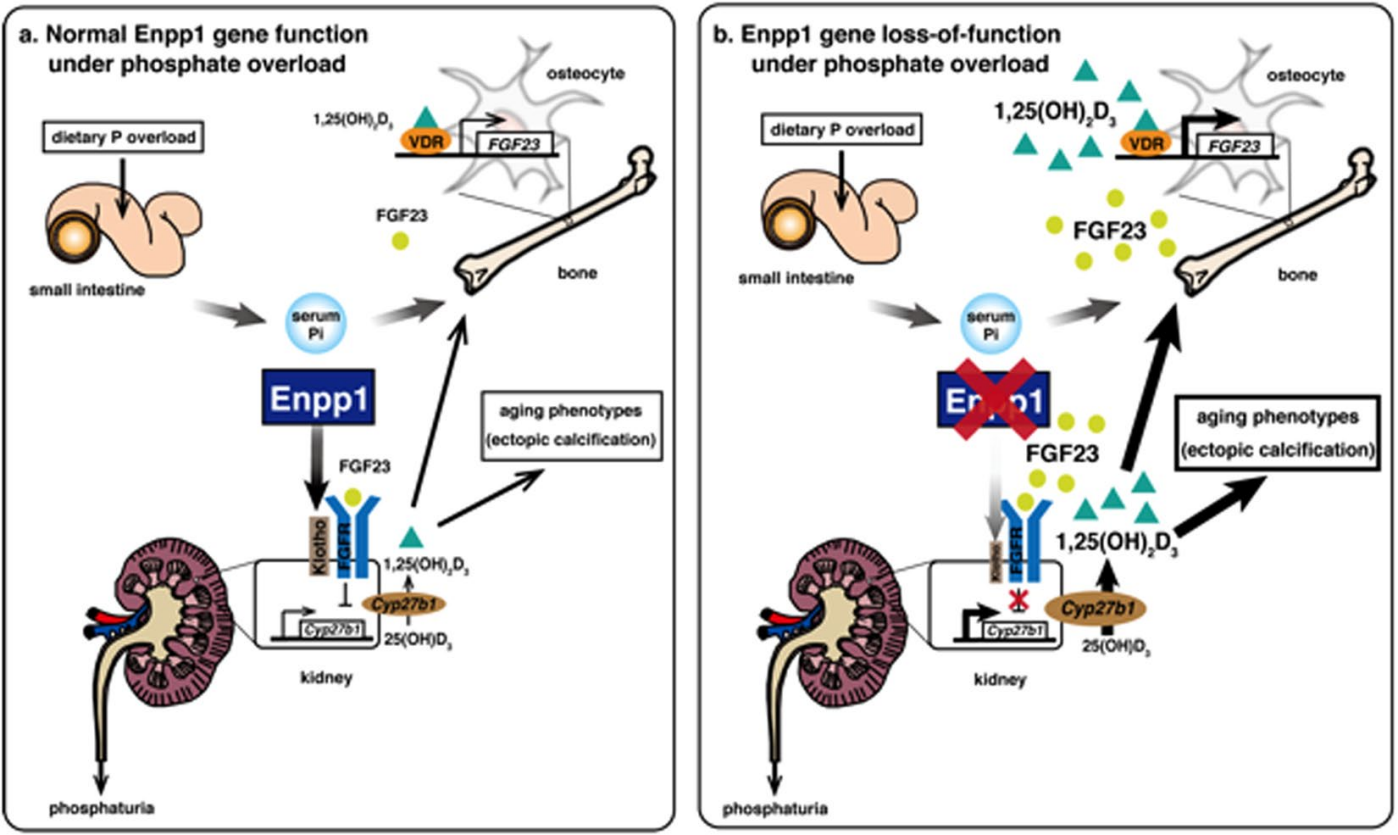
A schematic showing regulation of aging phenotypes under phosphate overload. (**a**) Normal Enpp1 maintains Klotho expression in kidney under dietary phosphate overload. Elevated serum phosphate levels promote FGF23 production from bone, and resultant FGF23 inhibits Cyp27 expression via a complex containing the FGF receptor (FGFR) and Klotho complex in kidney, inhibiting 1,25(OH)_2_D_3_ overproduction and suppressing aging phenotypes. (**b**) Enpp1 loss significantly downregulates renal Klotho expression under dietary phosphate overload. Elevated serum phosphate levels promote FGF23 production from bone, but the FGF23 signals are altered due to decreased Klotho expression. This outcome results elevates Cyp27b1 expression, in turn leading to 1,25(OH)_2_D_3_ overproduction and inducing aging phenotypes.

How to achieve longevity is a fundamental concern for people living all over the world. The development of strategies to promote healthy aging requires understanding molecular mechanisms underlying regulation of aging, and various molecules have been identified as aging-related^51–55^. Here, we show that under phosphate overload, Enpp1 is required for renal Klotho expression, and its activity leads to down-regulation of 1,25(OH)_2_D_3_ production by inhibiting cyp27b1 expression and is part of a crucial axis that regulates phosphate and vitamin D metabolism (Fig. 6a). Enpp1 is also required to suppress FGF23 production by osteocytes, and Enpp1 loss elevates serum FGF23 levels owing to increased serum 1,25(OH)_2_D_3_ (Fig. 6b). Thus Enpp1 serves as an upstream mediator of the active Klotho/vitamin D_3_/FGF23 axis to suppress aging phenotypes (Fig. 6).

Previous studies demonstrate that Klotho plays a pivotal role in regulating aging^2^, stimulating a search for upstream regulators of Klotho^56–58^. Promoter methylation has been demonstrated to restrict Klotho expression in the kidney^25^. However, the targeting of potential upstream regulators of Klotho has not yet yielded animal models that resemble *kl/kl* mice in terms of premature aging. Here, we demonstrate that mutation of Enpp1 phenocopies *kl/kl* mice under conditions of phosphate overload. How Enpp1 regulates Klotho expression in kidney is unclear, and this remains to be addressed. However, since Enpp1 is reportedly expressed in osteocytes^59^, Enpp1 expressed in those cells may regulate renal Klotho expression. Further studies are needed to define the regulatory system between bone and kidney in the context of aging. It is also possible that Klotho expression is inhibited by phosphate overload itself. Indeed, in our study *Klotho* expression was down-regulated by phosphate overload even in WT mice (Fig. 3d), although these changes were not statistically significant. Thus, low phosphate conditions caused by low vitamin D diet or VDR-deficiency likely inhibit Klotho down-regulation and contribute to reversal of aging phenotypes seen in *Enpp1* ^*ttw/ttw*^ mice fed a HPD. However, since *Klotho* expression was lower in *Enpp1* ^*ttw/ttw*^ than in WT mice, Enpp1 is likely required for *Klotho* expression. Nonetheless, VDR could be a therapeutic target to treat Enpp1-deficient patients.

Various *Enpp1* gene mutations have been identified in humans, but phenotypes of patients harboring differing *Enpp1* mutations vary from hypophosphatemia rickets or GACI to OPLL^29, 30, 34^. There are several types of hypophosphatemia rickets, among them XLH (*Phex* mutation), ARHR1 (*DMP1* mutation) and ARHR2 (*Enpp1* mutation). Currently, patients with any of these conditions are treated with vitamin D_3_ (calcitriol) and phosphorus supplementation. The effects of such treatment on ARHR2 patients reportedly varies, and some patients are reportedly worsened by treatment^60^. Here we propose that supplementation of patients harboring an *Enpp1* mutation with vitamin D_3_ (calcitriol) and phosphorus is a potential contraindication, and that by contrast, inhibition of vitamin D signals should be considered.

Among Enpp1 mutations, GACI is associated with the most severe phenotypes, and patients exhibit calcification of the aorta, with a mortality rate of approximately 85% by the age of 6 months^18^. GACI phenotypes are reminiscent of *Enpp1* ^*ttw/ttw*^ and *Enpp1* ^*Δ/Δ*^ mice fed a HPD or of *kl/kl* mice. To date, patients with GACI are treated with bisphosphonate^61, 62^. In animal models, Enpp1-Fc is reportedly effective in blocking HPD-induced death in Enpp1 mutant mice^63^. OPLL is a disease characterized by ossification of the PLL of the spine, although its molecular pathogenesis is unclear, and no therapeutic drugs have been established. Several gene mutations are reportedly associated with OPLL^64^, and *Enpp1* mutations are detected in some OPLL patients^34^.

Recent advances in aging research have revealed that excess dietary phosphate intake accelerates aging and renal dysfunction^65^. Klotho plays an essential role in regulating phosphate levels, and *kl/kl* mice exhibit various aging phenotypes seen in humans^44^. The Enpp1-Klotho/FGF23-VDR axis is considered crucial to regulate human aging by controlling circulating phosphate levels. Moreover, Klotho and Fetuin A, the latter a soluble protein produced in the liver, are reportedly required to regulate circulating phosphate levels, and Fetuin A-deficient mice exhibit systemic ectopic calcification^8^. Fetuin A reportedly forms “calciprotein particles” to inhibit precipitation of calcium and phosphate to prevent unwanted calcification^66^, and circulating Fetuin A levels are reportedly down-regulated with age^48^. Thus, aging and changes in phosphate levels are regulated in a complex manner, and further studies are needed to clarify mechanisms underlying their relationship.

Overall, our study sheds light on the pathogenesis of *Enpp1* mutation-related disease. We conclude that inhibition of the vitamin D3-VDR pathway may be a better option to treat patients with *Enpp1* mutations, although further clinical studies are required to test this strategy.

## Methods

### Mice and diets

The *Enpp1* ^*ttw/ttw*^ mouse is a spontaneous mutant harboring a nonsense mutation in *Enpp1* and first characterized in 1998 as an excellent model of ectopic ossification^38, 67^. We obtained *Enpp1* ^*wt/ttw*^ heterozygotes from the Central Institute for Experimental Animals (Kawasaki, Japan) and mated them to obtain *Enpp1* ^*ttw/ttw*^ homozygotes. Wild-type mice were obtained from Sankyo Lab Service (Tsuchiura, Ibaraki, Japan). Klotho-mutant (*kl/kl*)^2^, Klotho-overexpressing transgenic (Klotho Tg)^68^, and vitamin D receptor (VDR)-deficient (*VDR* ^*−/−*^) mice were established previously^69^. Klotho Tg or *VDR* ^*−/−*^ mice were crossed with *Enpp1* ^*ttw/ttw*^ mice to yield *Enpp1* ^*ttw/ttw*^/Klotho Tg or *Enpp1* ^*ttw/ttw*^ *VDR* ^*−/−*^ mice, respectively. Mice were fed either a normal phosphate diet (1% phosphate, ND), a HPD (1.5–2% phosphate, HPD), a high phosphate/low vitamin D (HPLD) diet or a high phosphate/high calcium diet starting at eight weeks of age for at least two weeks or indicated periods. An HPLD contains 0 units/100 g Vitamin D units. Other diets contain 240 units/100 g. Components of the HPD and HPLD are shown in the Supplementary Table 1. All animal methods were carried out in accordance with the Guidelines of the Keio University animal care committee. All experimental protocols were approved by that committee.

### Quantitative PCR Analysis

Total RNAs were isolated from kidney by TRIzol reagent (Invitrogen Corp.), and cDNA synthesis was done using oligo(dT) primers and reverse transcriptase (Wako Pure Chemicals Industries). Quantitative PCR was performed using SYBR Premix ExTaq II reagent and a DICE Thermal cycler (Takara Bio Inc., Otsu, Shiga, Japan). Samples were matched to a standard curve generated by amplifying serially diluted products using the same PCR reactions. *β-actin* (*Actb*) expression served as an internal control. Primers for *Klotho*, *Cyp27b1* and *Actb* were as follows.

*Klotho*-forward: 5′-GACAATGGCTTTCCTCCTTTACCT-3′

*Klotho*-reverse: 5′-TGCACATCCCACAGATAGACATTC-3′

*Cyp27b1*-forward: 5′-ACTCAGCTTCCTGGCTGAACTCTT-3′

*Cyp27b1*-reverse: 5′-GTAAACTGTGCGAAGTGTCCCAAA-3′

*Runx2*-forward: 5′-GACGTGCCCAGGCGTATTTC-3′

*Runx2*-reverse: 5′-AAGGTGGCTGGGTAGTGCATTC-3′

*β-actin (Actb)*-forward: 5′-TGAGAGGGAAATCGTGCGTGAC-3′

*β-actin (Actb)*-reverse: 5′-AAGAAGGAAGGCTGGAAAAGAG-3′

### Western blotting

Harvested kidneys were homogenized in RIPA buffer (1% Tween 20, 0.1% SDS, 150 nM NaCl, 10 mM Tris-HCl (pH 7.4), 0.25 mM phenylmethylsulfonyl fluoride, 10 g/ml aprotinin, 10 g/ml leupeptin, 1 mM Na3VO4, 5 mM NaF (Sigma)). Lysates were collected by centrifugation at 15,000 rpm at 4 °C for 10 min. Equivalent amounts of protein were separated by SDS/PAGE and transferred to a PVDF membrane (EMD Millipore Corporation). Proteins were detected using anti-Klotho (ab98111, abcam, Cambridge, UK), anti-NaPi-IIa (ab182099, abcam) and anti-Actin (A2066, Sigma) antibodies. Bands were visualized using ECL Western Blotting Detection Reagent (GE Healthcare, Uppsala, Sweden).

### Analysis of skeletal morphology

Bone mineral density (BMD) of whole femurs was measured by Dual-energy X-ray absorptiometry (DEXA) using a DCS-600R system (Aloka Co. Ltd., Tokyo, Japan). Vertebral bones and surrounding tissues including intervertebral discs and the posterior longitudinal ligament were scanned by a micro-computed tomography (R_mCT2; Rigaku Corp., Tokyo, Japan) at 90 kV and 160 A. Two-dimensional regions of interest were created at the level of the cervical spine and Achilles tendon using TRI/3D-BON software (RATOC Co. Ltd., Tokyo, Japan).

### Histopathological analysis

Kidney, aorta and skin from euthanized mice were fixed in 10% phosphate-buffered formalin, and embedded in paraffin. Tissues were sectioned and stained with hematoxylin and eosin (H&E) and using von Kossa methods. Slides were examined by light microscopy (BIOREVO, BZ-9000 (Keyence, Osaka, Japan)) for tissue mineralization. Relative calcification areas in kidney and aorta were calculated using microscopic image analysis software BZ-II analyzer (Keyence). Kidney sections were also stained using anti-p16 INK4A (10883-1-AP, Proteintech, Rosemont, IN, USA) or anti-Klotho (ab98111, abcam) followed by Alexa488-conjugated goat anti-rabbit IgG H&L (Alexa Fluor^®^ (ab150077, abcam), and nuclei were stained with DAPI (Wako Pure Chemicals Industries, Osaka, Japan). Senescence-associated beta-galactosidase was stained using a SA-β-gal kit (#9860, Cell Signaling, Danvers, MA). Then, sections were observed under a fluorescence microscope (Keyence, Osaka, Japan).

### ELISA

Serum levels of FGF23 (full length, KAINOS Lab Inc., Tokyo, Japan), RANKL (R&D, systems, Inc., Minneapolis, MN), OPG (R&D) CTx (Immunodiagnostic Systems Limited, Boldon, UK), urinary Fetuin A (R&D) and α-Klotho (Immuno-Biological Laboratories Co, Ltd, Gunma, Japan) were measured by using an ELISA kit based on the manufacturers’ instructions.

### Biochemical analyses

Peripheral blood was obtained from the postorbital vein. Serum was isolated by centrifugation at 6,000 rpm for 15 minutes at 4 °C and stored at −80 °C. 1,25(OH)2D3 and 25(OH)D3 levels in sera were measured using a 25OH-Vitamin D total-RIA-CT kit (DIAsource, Ottignies-Louvain-la-Neuve, Belgium) and an ECLIA kit (Cobas, Roche Diagnostics, Basel, Switzerland), respectively.

### Statistical analysis

Data were analyzed using a two-tailed Student’s *t*-test. For all graphs, data are represented as means ± standard deviation (SD). A *p*-value less than 0.05 was considered statistically significant (*p < 0.05; **p < 0.01; ***p < 0.001).

## Electronic supplementary material

Supplementary Figures
