# Supplementary material for: Enpp1 is an anti-aging factor that regulates Klotho under phosphate overload conditions
Source: Sci Rep. 2017 Aug 10;7:7786. doi: 10.1038/s41598-017-07341-2 (PMC5552841; doi:10.1038/s41598-017-07341-2)
Supplement: Supplementary file 1 — Supplementary Figures [file 41598_2017_7341_MOESM1_ESM.pdf]

Enpp1 is an anti-aging factor that regulates Klotho under phosphate overload conditions

Ryuichi Watanabe[1], Nobuyuki Fujita[1], Yuiko Sato[1,2], Tami Kobayashi[1,3], Mayu Morita[4], Takatsugu Oike[1], Kana Miyamoto[1], Makoto Kuro-o[5], Toshimi Michigami[6], Seiji Fukumoto[7], Takashi Tsuji[1], Yoshiaki Toyama[1], Masaya Nakamura[1], Morio Matsumoto[1] and Takeshi Miyamoto[1,2]

[1]Department of Orthopedic Surgery, [2]Department of Advanced Therapy for Musculoskeletal Disorders, [3]Department of Musculoskeletal Reconstruction and Regeneration Surgery, [4]Division of Oral and Maxillofacial Surgery, Department of Dentistry and Oral Surgery, Keio University School of Medicine, 35 Shinano-machi, Shinjuku-ku, Tokyo 160-8582, Japan; [5]Center for Molecular Medicine, Jichi Medical University, Shimotsuke, Tochigi 329-0498, Japan; [6]Department of Bone and Mineral Research, Osaka Medical Center and Research Institute for Maternal and Child Health, Izumi, Osaka 594-1101, Japan; [7]Fujii Memorial Institute of Medical Sciences, Tokushima University, Tokushima, Tokushima 770-8503, Japan

Supplementary Figure Legends

Supplementary Figure 1. Ectopic calcification seen in *Enpp1*$^{ttw/ttw}$ mice is due to trans-differentiation of cells into osteoblastic lineage.

(a) Representative histopathology of the ectopic calcification by von Kossa staining in the kidney and aorta in *Enpp1*$^{ttw/ttw}$ mice fed a HPD. Bar=100 μm (a) *Runx2* expression in the aorta of *Enpp1*$^{ttw/ttw}$ or wild-type (WT) mice fed either a ND or HPD by realtime PCR (b). Data in (b) represent *Enpp1* expression relative to *β-actin* ± SD (**, $p < 0.01$; ***, $p < 0.001$; $n = 5$).

Supplementary Figure 2. p16 is expressed in the kidney of *Enpp1*$^{ttw/ttw}$ mice fed a HPD.

Expression of p16 or SA *β*-gal in kidney of *Enpp1*$^{ttw/ttw}$ mice or wild-type (WT) mice fed either ND or HPD was analyzed by immune-histochemical staining. Nuclei were stained with DAPI. Bar=100 μm.

Supplementary Figure 3.

Decreased bone mass in *Enpp1*$^{ttw/ttw}$ mice fed a HPD is likely due to elevated osteoclast bone-resorption.

Serum levels of RANKL (a), OPG (b) and CTX-I (d) in *Enpp1*$^{ttw/ttw}$ mice and wild-type (WT) mice fed either ND or HPD were analyzed by ELISA. RANKL/OPG ratio is

calculated by the results of (a) and (b). All Data represent mean indicated parameter ± S.D. (#, $p < 0.\ 1$; *, $p < 0.05$; ***, $p < 0.001$; *ns*, not significant; each group; n=5).

Supplementary Figure 4. Aggravated renal function in *Enpp1*$^{ttw/ttw}$ mice by dietary phosphate overloading.

Serum levels of BUN and creatinine (a), urine volume collected over 24-hours collection (b) and urinary levels of creatinine, calcium and phosphorus (c) in *Enpp1*$^{ttw/ttw}$ mice and wild-type (WT) mice with either ND or HPD diet are shown. All Data represent mean values of the indicated parameter ± SD (#, $p < 0.1$; *, $p < 0.05$; ***, $p < 0.001$; *ns*, not significant; each group; n=5).

Supplementary Figure 5. Dietary phosphate overload decreases Klotho expression in kidney of *Enpp1*$^{ttw/ttw}$ mice.

Eight-week-old wild-type and *Enpp1*$^{ttw/ttw}$ mice were fed a ND or HPD for two weeks, and Klotho, NaPi-IIa and Actin expression in kidney was analyzed by western blot. Representative data of at least three independent experiments are shown.

Supplementary Figure 6. Klotho overexpression in *Enpp1*$^{ttw/ttw}$ mice alleviates the aging phenotypes

Eight weeks old wild-type (WT), Klotho transgenic (*Kl Tg*), *Enpp1*$^{ttw/ttw}$, *Kl Tg/Enpp1*$^{ttw/ttw}$ mice were fed with HPD for indicated period (a) or two weeks (b). Then, changes in survival rate was monitored (a), ectopic calcification in kidney and aorta was

analyzed by von Kossa staining (b, left panel) and the calcification area in aorta was scored (b, right panel). Data (b) represent mean ectopic calcification area ± S.D. (***, $p < 0.001$, $n = 6$).

Supplementary Figure 7. Ectopic calcification of intervertebral disc in *kl/kl* mice represent OPLL-like phenotypes in *Enpp1*$^{ttw/ttw}$ mice

The representative images of ectopic calcification around the vertebral bones in six weeks old wild-type (WT) and *Klotho* gene mutant mice (*kl/kl*) was analyzed by micro-computed tomography.

Supplementary Figure8. Urinary measurements in WT and *Enpp1*$^{ttw/ttw}$ mice fed a HPLD.

Urinary levels of creatinine, calcium and phosphorus are measured in *Enpp1*$^{ttw/ttw}$ or wild-type (WT) mice fed either ND, HPD or HPLD are shown. All Data represent mean values of the indicated parameter ± SD (#, $p < 0.1$; *, $p < 0.05$; **, $p < 0.01$; ***, $p < 0.001$; *ns*, not significant; each group; n=5).

Supplementary Figure9. Fetuin A levels are not associated with aging phenotypes seen in *Enpp1*$^{ttw/ttw}$ mice fed a ND, HPD or HPLD.

Serum Fetuin A levels in were measured by ELISA in *Enpp1*$^{ttw/ttw}$ mice and wild-type (WT) mice fed either ND, HPD or HPLD. Data represent mean urine Fetuine A concentration ± SD (*, $p < 0.05$; *ns*, not significant; each group; n=5).

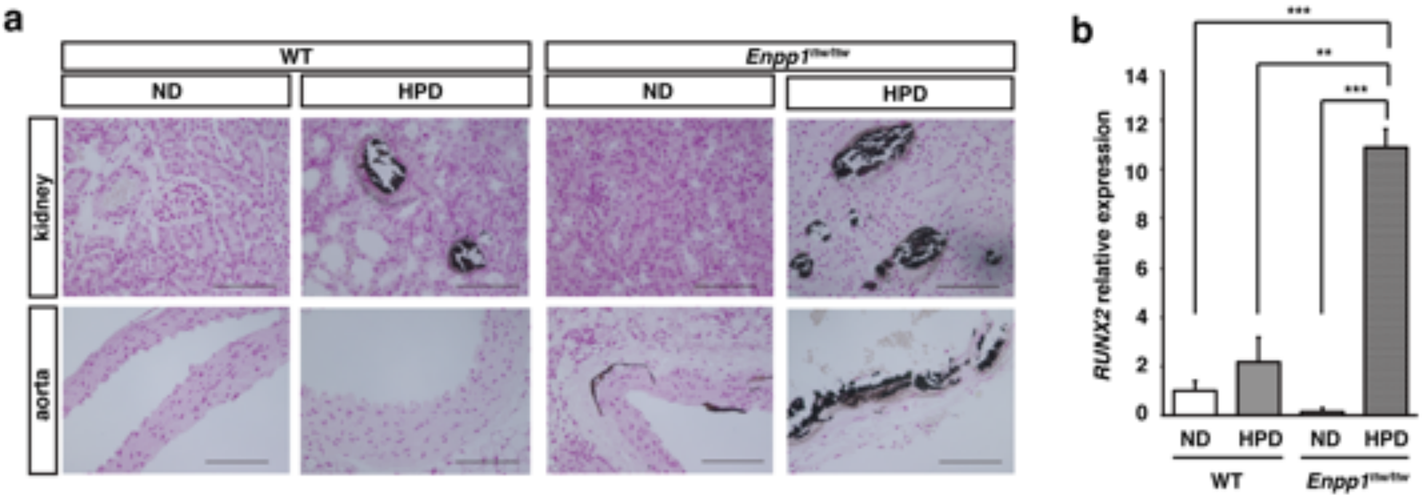

Figure S1. Watanabe R, et al.

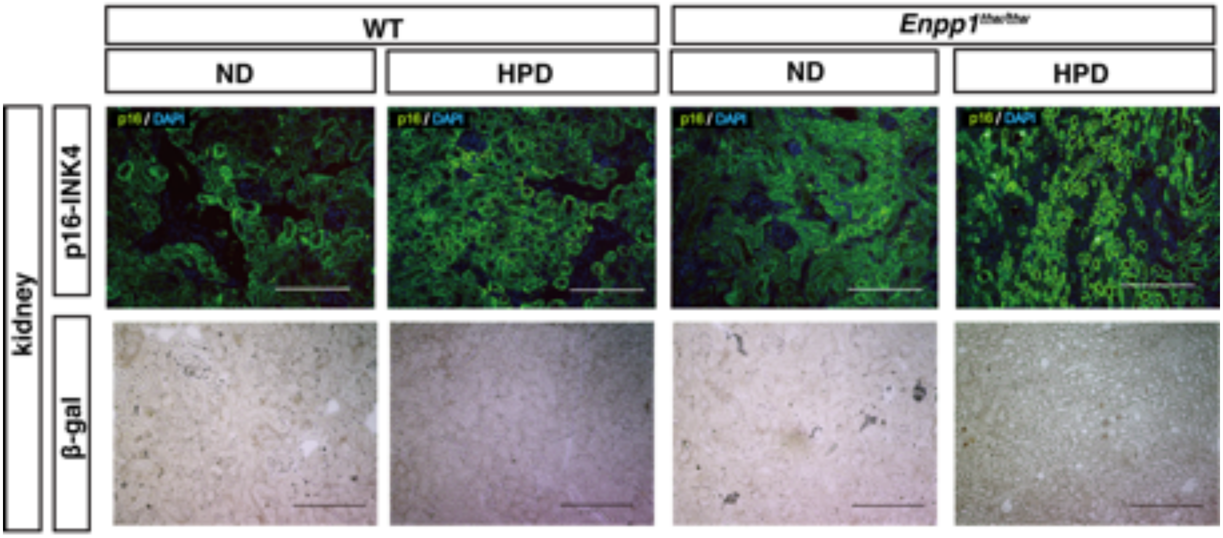

Figure S2. Watanabe R, et al.

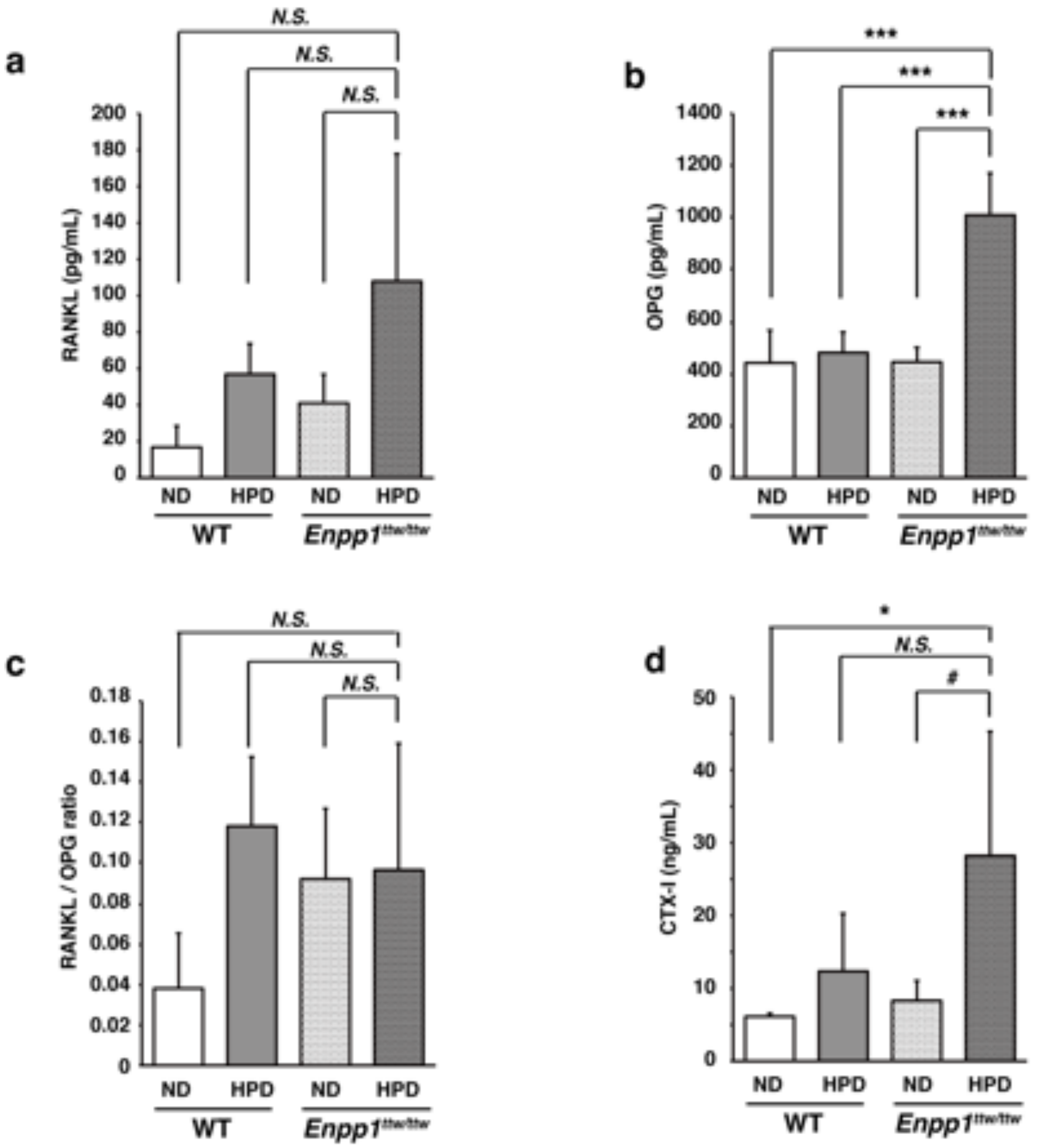

Figure S3. Watanabe R, et al.

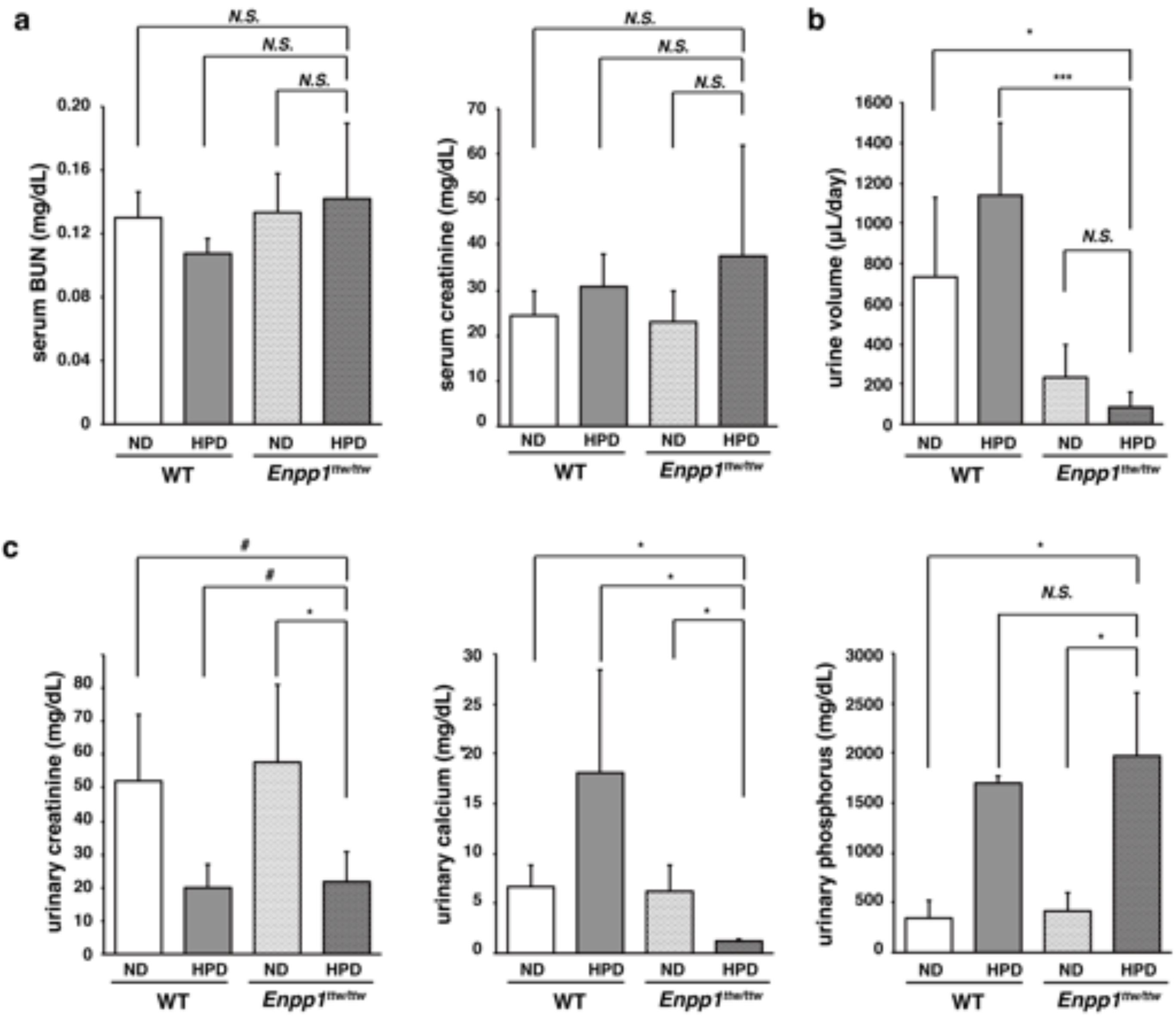

Figure S4. Watanabe R, et al.

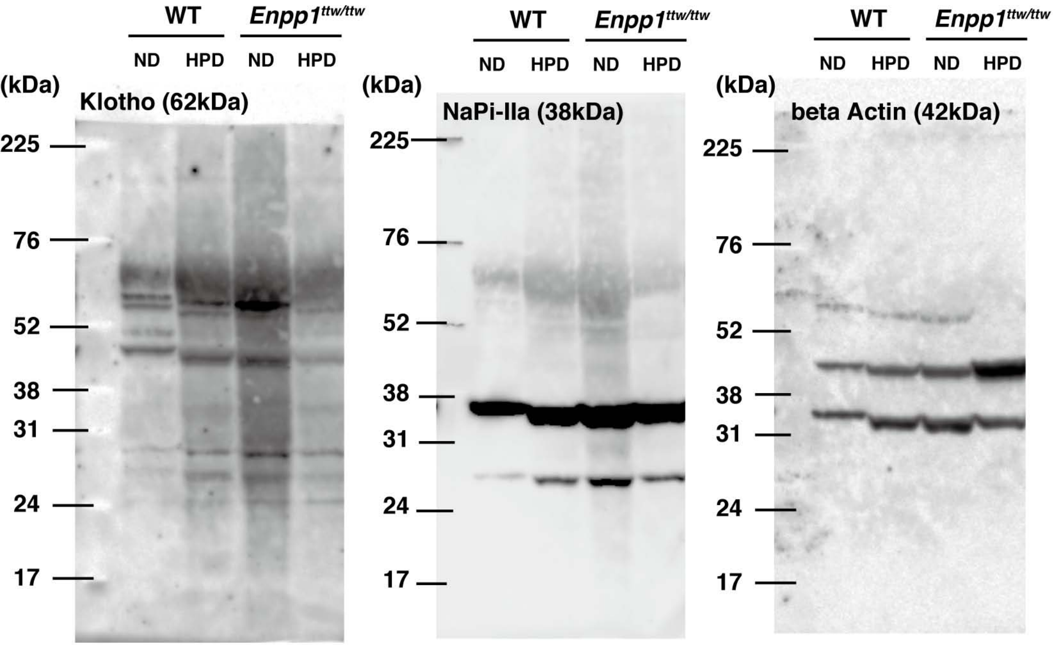

FigureS5. Watanabe R, et al.

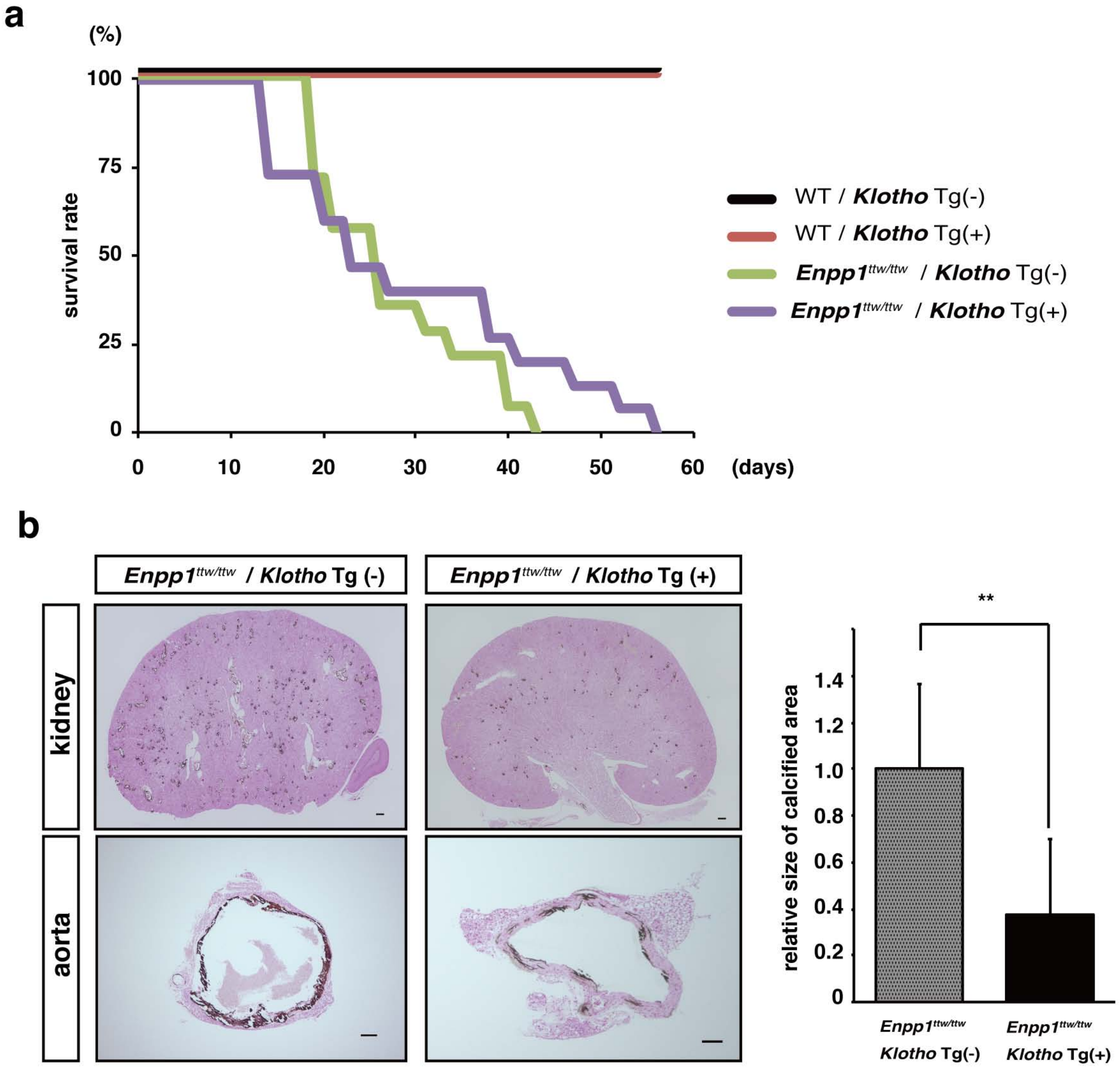

FigureS6. Watanabe R, et al.

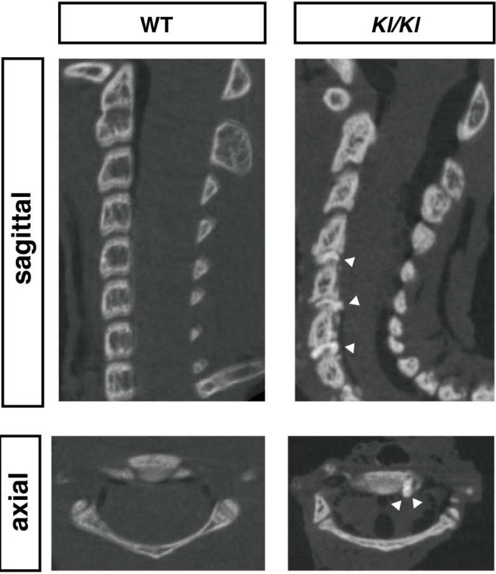

FigureS7. Watanabe R, et al.

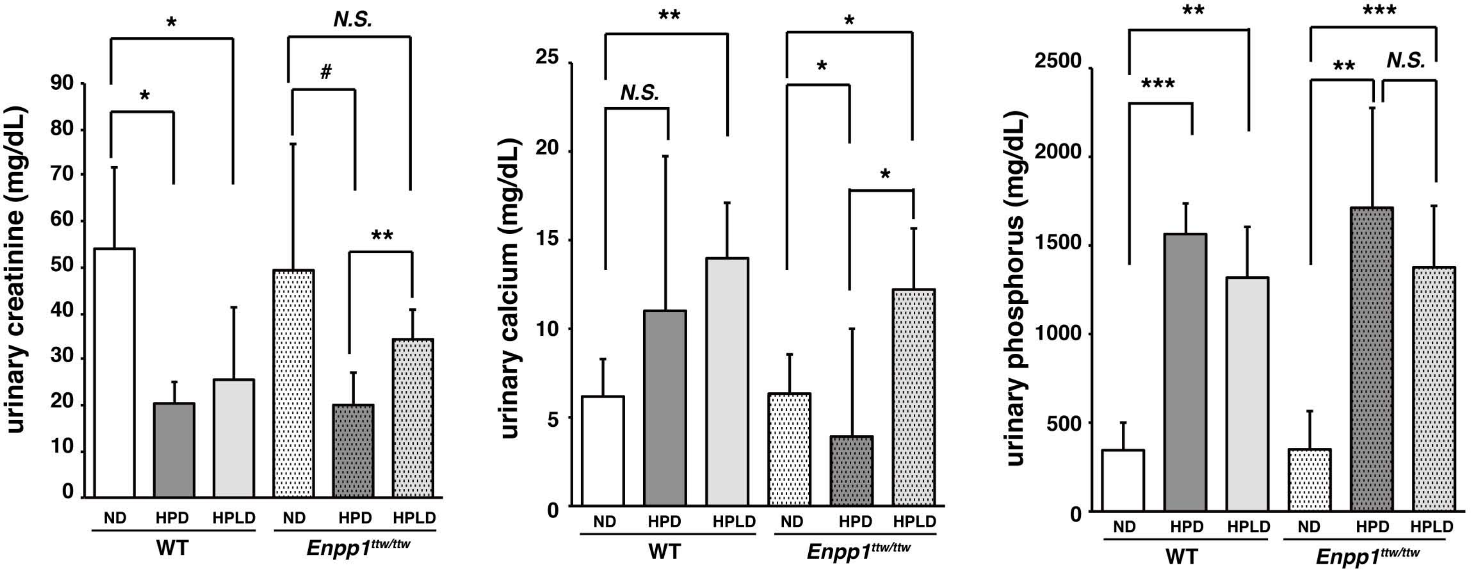

Figure S8. Watanabe R, et al.

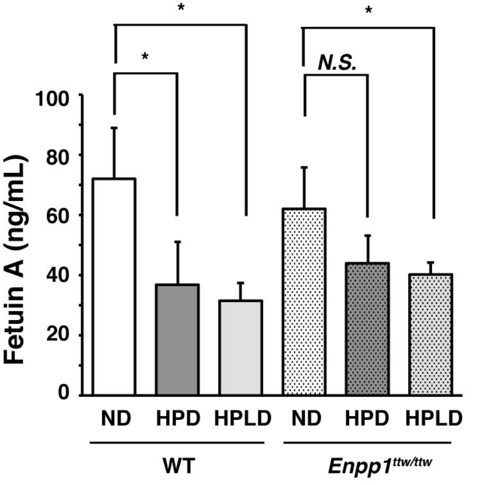

Figure S9. Watanabe R, et al.
